# Sex and age differences in mortality trends of gastric cancer among Hispanic/Latino populations in the United States, Latin America, and the Caribbean

**DOI:** 10.1016/j.lana.2022.100376

**Published:** 2022-10-07

**Authors:** J. Smith Torres-Roman, Christian S. Alvarez, Pedro Guerra-Canchari, Bryan Valcarcel, José Fabián Martinez-Herrera, Carlos A. Dávila-Hernández, Camila Alves Santos, Samara Carollyne Mafra Soares, Dyego Leandro Bezerra de Souza, M. Constanza Camargo

**Affiliations:** a Universidad Científica del Sur, Lima, Peru; b Latin American Network for Cancer Research (LAN–CANCER), Lima, Peru; c Division of Cancer Epidemiology and Genetics, National Cancer Institute, Rockville, Maryland, USA; d Sociedad Científica San Fernando, Universidad Nacional Mayor de San Marcos, Lima, Peru; e Cancer Center, Medical Center American British Cowdray, Mexico City, Mexico; f Universidad Privada San Juan Bautista, Ica, Peru; g Graduate Program in Collective Health, Federal University of Rio Grande do Norte, Natal, Rio Grande do Norte state, Brazil; h Research group on Methodology, Methods, Models and Outcomes of Health and Social Sciences, School of Health Sciences and Welfare, Centre for Health and Social Care Research, University of Vic-Central University of Catalonia, Spain

**Keywords:** Gastric cancer, Hispanics, Latinos, Mortality trends

## Abstract

**Background:**

An up-to-date analysis of gastric cancer mortality among Hispanic/Latino populations is required for estimating disease burden and assessing the effectiveness of clinical and preventive strategies.

**Methods:**

We retrieved gastric cancer deaths between 1997 and 2017 (as available) from the Surveillance, Epidemiology, and End Results Program (United States Hispanics) and the World Health Organization databases (Puerto Rico, 16 Latin American and Caribbean countries). Joinpoint regression analysis was used to examine trends in age-standardized mortality rates (ASMR; per 100 000 person-years) and calculate average annual percent changes (AAPCs) by country (or territory), age group (25–49 and ≥50 years), and sex. Trends were compared to assess slope parallelism.

**Findings:**

In 2017, Chile (31·8), Colombia (24·3) and Costa Rica (24·3) had the highest ASMR of gastric cancer for men, while Guatemala (17·2), Peru (13·5), and Costa Rica (13·3) had the highest ASMR for women. Small-to-moderate mortality declines (AAPCs ranged −4 to −0.5%) were observed between 1997 and 2017. In almost all countries, trends decreased among individuals aged ≥50 years. However, age-specific trends were not parallel (p-values <0.05) in Brazil, Colombia, Mexico, the United States, and Venezuela for both men and women, and in five additional countries for only women; with a few countries showing stable or slightly increasing trends for individuals aged 25–49 years.

**Interpretation:**

Overall gastric cancer mortality rates in Hispanics/Latinos declined in the last two decades. However, there was a notable variation in trends by country, sex, and age group. Continued and targeted prevention efforts are needed to reduce the disease burden in these vulnerable populations.

**Funding:**

Universidad Cientifica del Sur, Peru, and National Cancer Institute, United States.

**Research in context**

**Evidence before this study**

Gastric cancer is one of the leading causes of cancer mortality globally, affecting disproportionally Hispanic/Latino populations. Coverage and quality of cancer incidence registration vary across geographic regions, with only ∼20% of the Latin American population covered by population-based registration. In most of the Americas, gastric cancer incidence rates are similar to mortality rates due to the lack of screening and limited successful treatment options. Temporal trends of mortality are important indicators of health progress. Previous studies of gastric cancer mortality trends in Hispanic/Latino populations have evaluated a limited number of populations and short periods of time.

**Added value of this study**

We used data from the Surveillance, Epidemiology, and End Results Program and the World Health Organization to provide the most up-to-date estimates on gastric cancer mortality trends at a country-specific level for Hispanic/Latino populations in the United States, Latin America, and the Caribbean. We present sex- and age-specific mortality rates, as well as trends from 1997 to 2017. We also predicted the burden of gastric cancer deaths by 2030. Although overall mortality rates are declining, the magnitude of change varies across Hispanic/Latino populations. Notably, over the last two decades, mortality rates of gastric cancer have been stable or increasing in a few young populations, particularly women.

**Implications of all the available evidence**

A comprehensive gastric cancer control program requires substantial public health measures, and possibly screening and surveillance by use of upper gastrointestinal endoscopy in certain high-risk populations. Gastric cancer is and will continue to be one of the top health concerns for several Hispanic/Latino populations. This study provides a baseline for the evaluation of future activities as decision-makers design interventions to reduce the mortality of gastric cancer in the region. In addition, further research into the causes of gastric cancer and its evolving nature in these populations is warranted.

Alt-text: Unlabelled box

## Introduction

Gastric cancer incidence and mortality rates have been declining over the last 30 years.^1^ Nevertheless, this neoplasia is currently the fourth leading cause of cancer death worldwide, after lung, colorectal, and liver cancers.^2^ Gastric cancer risk widely varies by geographical region,^1^ with Eastern Asia exhibiting the highest rates worldwide. The substantial global reduction in gastric cancer incidence and mortality is attributed to decreasing prevalence of *Helicobacter pylori* infection and other well-recognized risk factors (i.e., smoking and high salt intake), as well as the implementation of successful screening programs in high-risk East Asian countries.^3^

Important advances have been made in cancer registration in Latin America. However, the proportion of the population covered by the existing cancer registries in the region is ∼20%, with high-quality information coverage estimated at 7·1%.^4^ In the absence of prevention strategies, gastric cancer is a lethal disease. In most of the Americas, gastric cancer incidence and mortality rates are similar because of lack of screening and limited successful treatment options. In the Latin American region, the mortality burden of gastric cancer in men aged 25 years or older represents 81% of its estimated incidence (age-adjusted rates: 21 *vs*. 17 per 100 000 population, respectively; GLOBOCAN 2020). In the United States, racial/ethnic minority populations, including Hispanics/Latinos have an increased risk of developing and dying from gastric cancer as compared to non-Hispanics whites.^5^ Latin America and the Caribbean is a region with low-to-moderate burden of gastric cancer. Previous studies of incidence and mortality of gastric cancer in Hispanic/Latino populations have been limited to short periods of time or a restricted number of countries.^6^, ^7^, ^8^ Temporal trends in disease mortality are key indicators of health system performance and may guide decision-making regarding public health interventions. Therefore, our study aimed to evaluate gastric cancer national mortality trends over 20 years for several Hispanic/Latino populations in the United States, Latin America and the Caribbean, and predict their future burden.

## Methods

### Population and setting

We used mortality data from the Surveillance, Epidemiology, and End Results (SEER) Program database for the United States (Hispanic/Latino populations only) and from the World Health Organization database for Puerto Rico (territory of the United States) and 16 Latin American and Caribbean countries. We retrieved mortality data for the period 1997 (or the first year available) to 2017 (or the last year available). The SEER database was accessed through SEER*Stat software (version 8.3.9.2), and the data were suppressed by default where counts were fewer than ten. Deaths from gastric cancer were identified with the code C16 (malignant neoplasm of the stomach) of the International Classification of Diseases 10^th^ revision. Our analysis was restricted to individuals diagnosed at ages 25 years or older. The thirteen 5-year age groups (i.e., 25-29, …, 80-84, and ≥85) were further collapsed into two groups, 25-49 and ≥50 years. Projected population estimates (medians) of the study countries (or territories) were obtained from the United Nations World Population Prospects database. Institutional review board approval was not applicable for this study.

### Statistical analyses

We estimated annual age-standardized mortality rates (ASMRs) per 100 000 person-years for 21 years (1997 to 2017) by the direct method using Segi's world standard population. We calculated male-to-female ratios of ASMRs for 1997 and 2017, and tested if there was a statistically significant difference between both years by Z test statistic. Age- and sex-specific mortality trends by country were estimated using Poisson models with the Joinpoint Regression Program version 4.8.0.1. We estimated country-specific average annual percentage change (AAPC) and the corresponding 95% confidence intervals (CI). AAPCs were considered statistically significant at a p-value <0.05. We performed pairwise tests of parallelism (based on segmented line regression models) to compare trends by age group (25–49 *vs*. ≥50 years) and sex (men *vs*. women). A set of two trends was considered not parallel if the p-value was <0.05.

Mortality rate predictions through 2030 were computed using the Nordpred package in R software.^9^ Briefly, an age-period-cohort model was fitted to the observed data (2003–2017), and then the Segi world-standardized mortality rates were calculated for the thirteen five-year age groups (i.e., 25–49, …, 80–84, ≥85), further collapsed into two groups (i.e., 25–49 and ≥50 years). An extrapolation of the trends based on the observed rates (i.e., 2003–2007, 2008–2012, and 2013–2017) was carried out for three 5-year calendar periods (i.e., 2018–2022, 2023–2027, and 2028–2032). For the predicted period, we used the median population of the year representing half of the five-year periods (i.e., 2020, 2025, and 2030). The method of cut trend was used to attenuate the linear drift by 0%, 25%, and 50%, at the first to third 5-year periods, respectively. The estimates for the year 2030 were calculated as the middle point for the last period 2028–2032. The numbers of predicted deaths due to changes in demographics (i.e., population growth and aging) were calculated using the observed rates, and the numbers predicted due to risk change were estimated as the difference between the total and those due to demographic changes.

### Role of the funding source

The funders of the study had no role in study design, data collection, data analysis, data interpretation, writing of the report, or the decision to publish.

## Results

In the 18 countries (or territories) included in this analysis, men had higher ASMRs than women. The male-to-female ratios for 2017 (or the most recent year) ranged from 1·1 in Guatemala to 2·7 in Chile (Supplementary Table 1).

**Figure 1:**
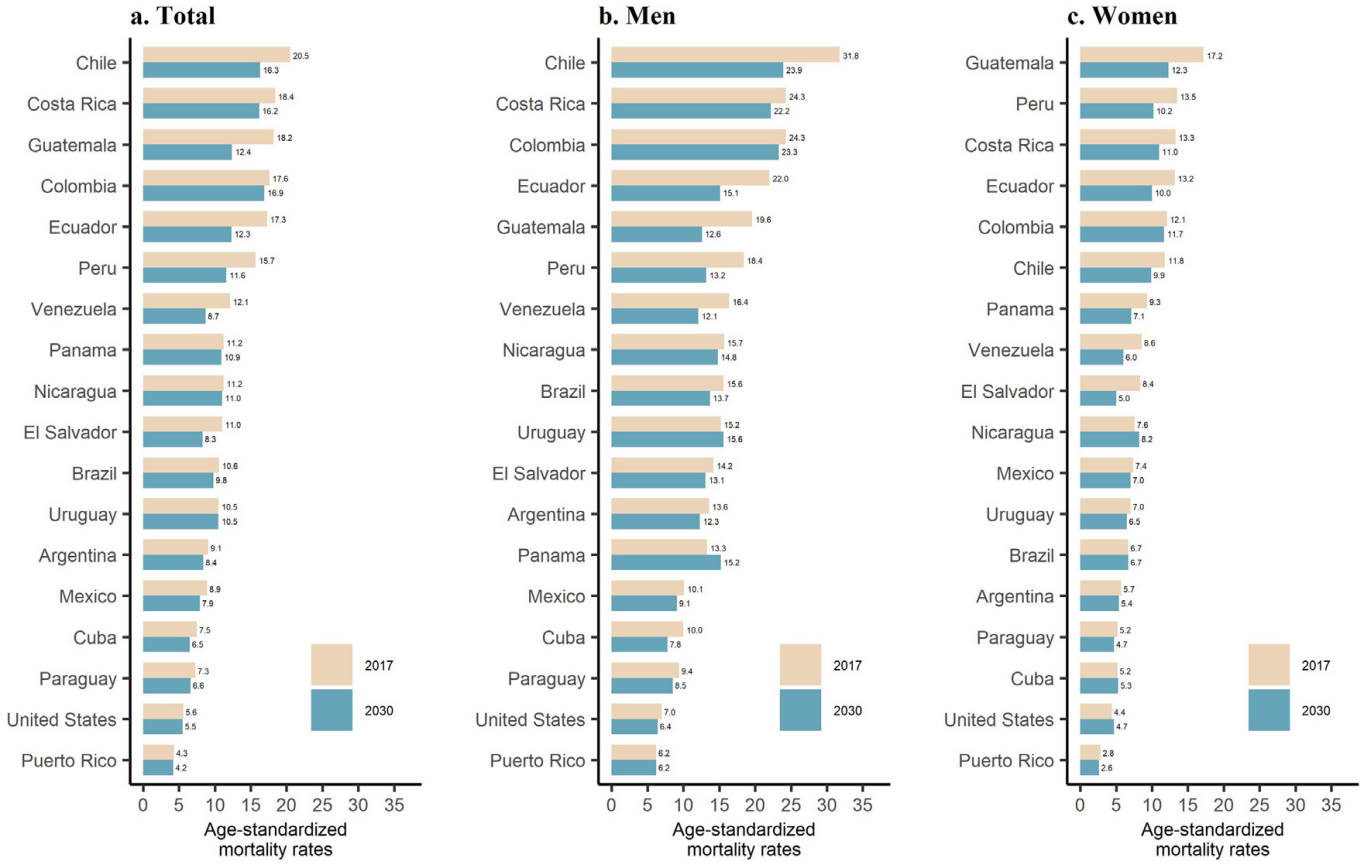
Age-standardized (SEGI's world standard population) gastric cancer mortality rates per 100 000 persons-years in 2017* and predicted 2030** among Hispanic/Latino populations aged 25 and older in the United States, Latin America, and the Caribbean. *Data from 2014 for Venezuela and 2015 for El Salvador; **Age-standardized rate represents the mid-point for the projected period 2028–2032.

Among men, Puerto Rico (6·2 per 100 000), the United States (7·0), and Paraguay (9·4) had the lowest ASMR in 2017, while Chile (31·8), Costa Rica (24·3), and Colombia (24·3) had the highest rates. Most countries are expected to experience a decrease in their mortality rates by 2030 (Figure 1). Among women, the lowest ASMRs in 2017 were observed in Puerto Rico (2·8 per 100 000), the United States (4·4), Paraguay (5·2), and Cuba (5·2), while the highest were in Guatemala (17·2), Peru (13·5), and Costa Rica (13·3).

**Figure 2:**
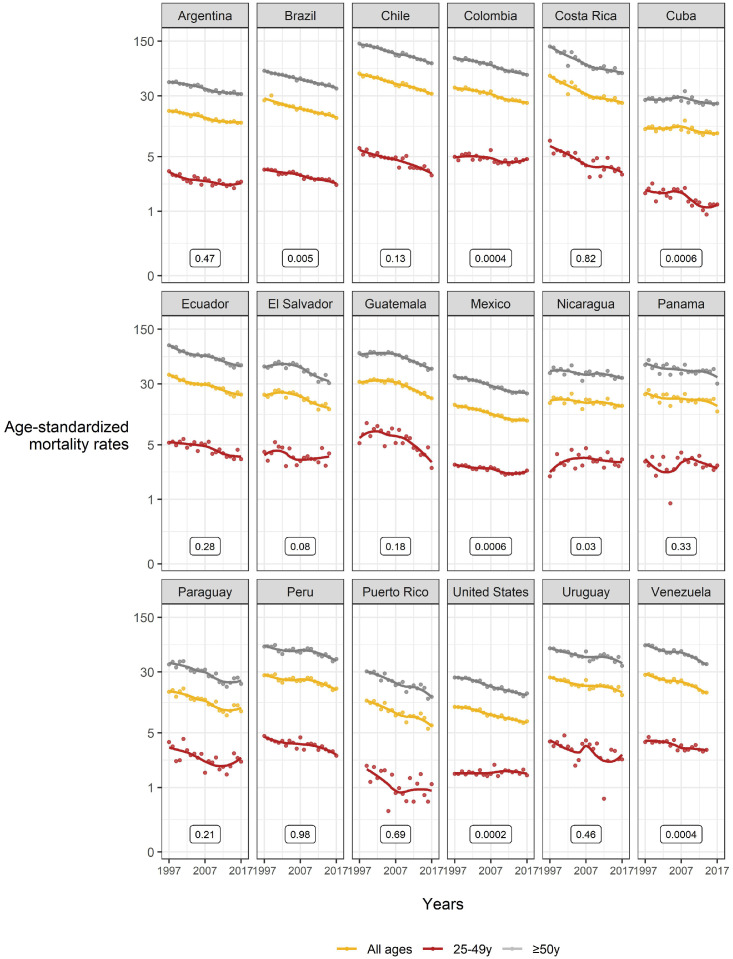
Gastric cancer mortality trends (rates per 100 000 persons-years; all ages, 25-49, and ≥50 years) 1997*-2017** among Hispanic/Latino males in the United States, Latin America, and the Caribbean. *Data from 1999 for Puerto Rico; **Data from 2014 for Venezuela and 2015 for El Salvador. *P*-values represent the difference for statistical parallelism tests (based on segmented line regression models) between 25–49 years and ≥50 years.

**Figure 3:**
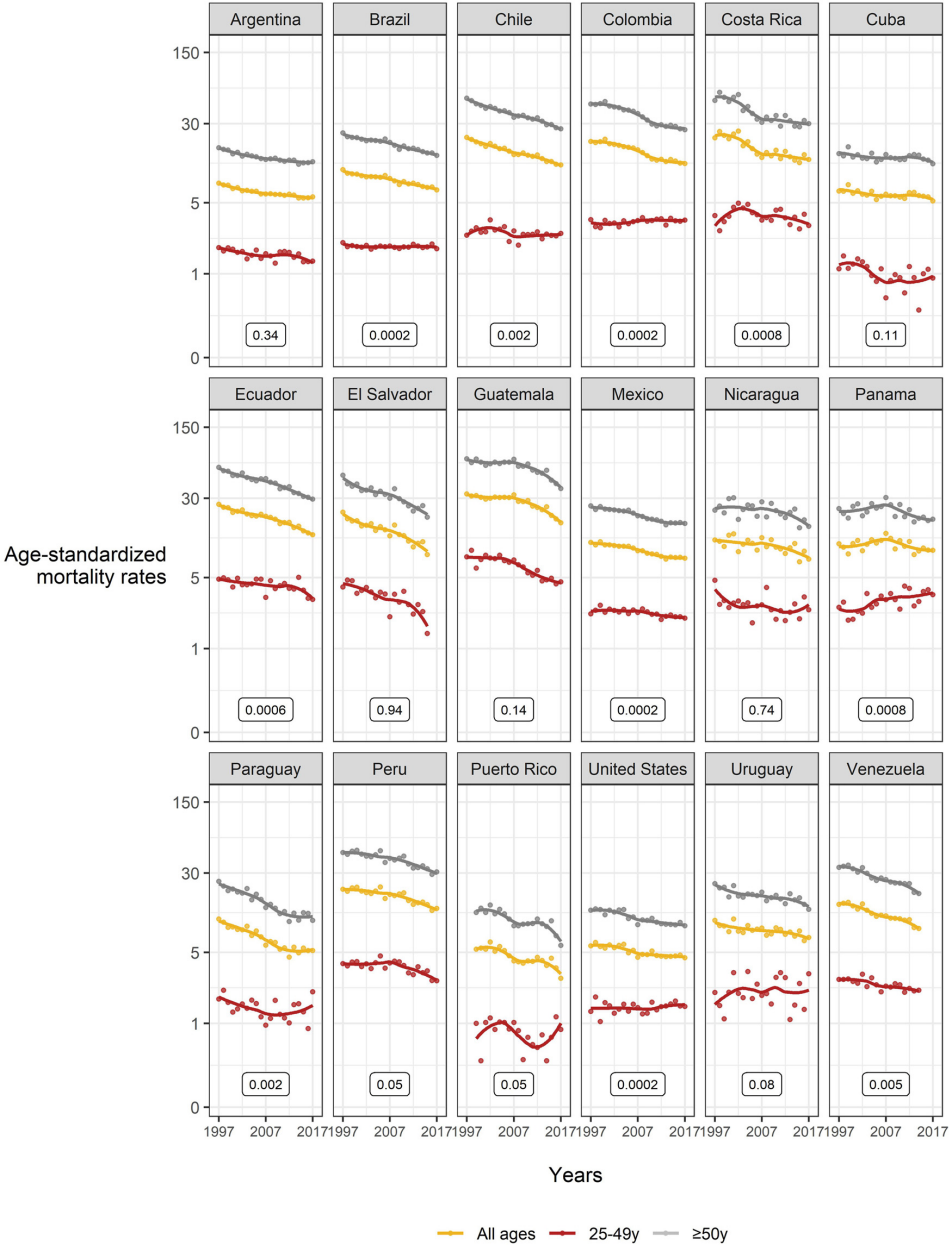
Gastric cancer mortality trends (rates per 100 000 persons-years; all ages, 25–49, and ≥50 years) 1997–2017 among Hispanic/Latino females in the United States, Latin America, and the Caribbean. *Data from 1999 for Puerto Rico; **Data from 2014 for Venezuela and 2015 for El Salvador. *P*-values represent the difference for statistical parallelism tests (based on segmented line regression models) between 25–49 years and ≥50 years.

Figures 2 and 3 show the overall and age-specific 1997-2017 mortality trends of gastric cancer for men and women, respectively. For both sexes, we observed downward trends in the overall population and the older age group (≥50 years). On the contrary, ASMRs in the young group (25–49 years) were less precise with a few countries showing stable or slightly increasing trends. In men (Figure 2), age-specific trends were not parallel in Brazil, Colombia, Cuba, Mexico, Nicaragua, the United States, and Venezuela. In women (Figure 3), age-specific trends were not parallel in Brazil, Chile, Colombia, Costa Rica, Ecuador, Mexico, Panama, Paraguay, the United States, and Venezuela.

**Figure 4:**
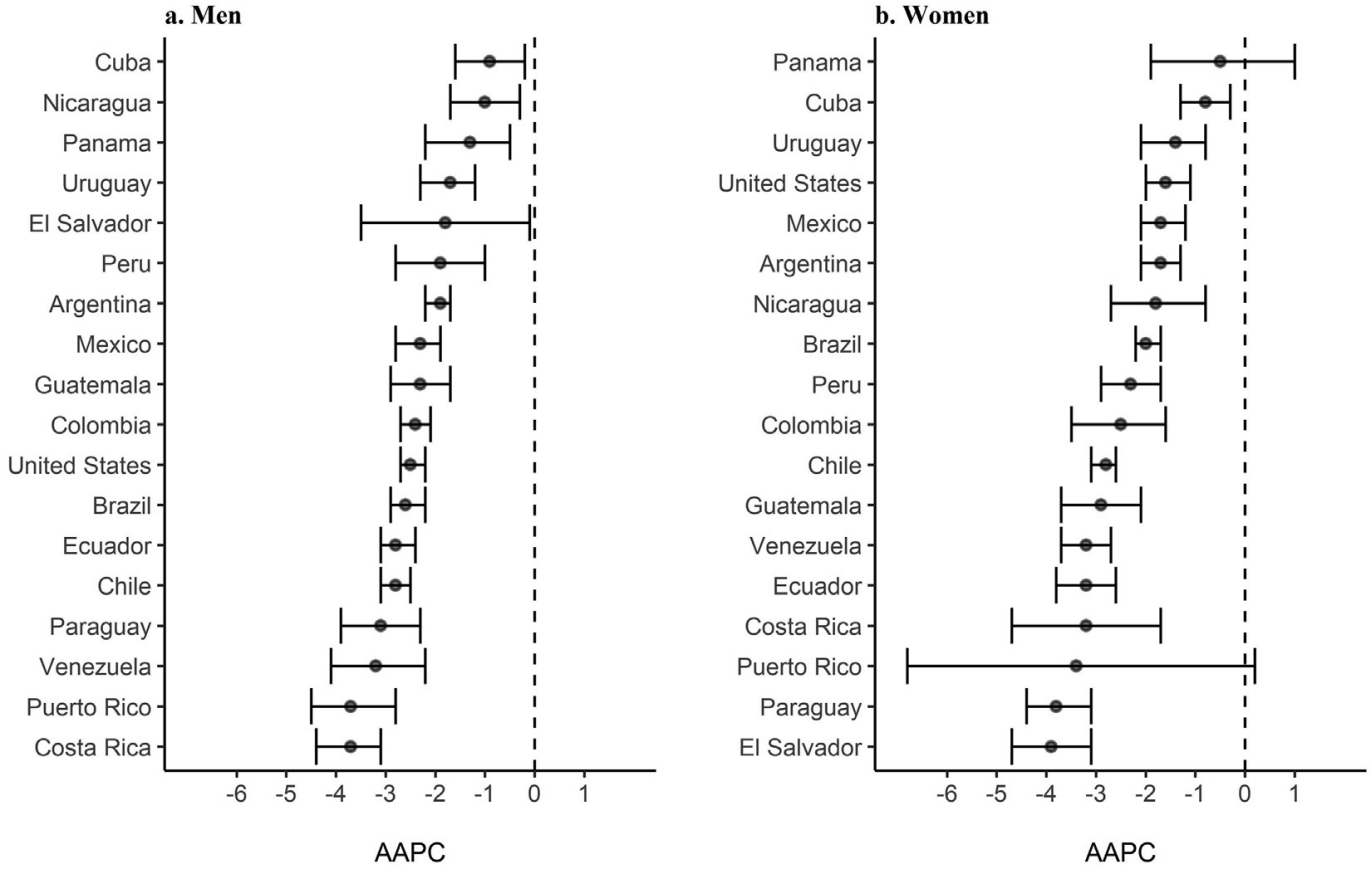
Average annual percent change (AAPC) and 95% confidence interval (CI) for gastric cancer age-adjusted mortality rates among Hispanic/Latino populations in the United States, Latin America, and the Caribbean by sex, 1997*–2017**. *Data from 1999 for Puerto Rico; **Data from 2014 for Venezuela and 2015 for El Salvador.

Regarding the overall (all ages) AAPC, all countries exhibited downward trends for both sexes (Figure 4 and Supplementary Table 1). In men, the countries with the highest changes were Costa Rica and Puerto Rico (AAPC, −3·7% for both countries), while those with the smallest changes were Cuba (AAPC, −0·9%) and Nicaragua (−1·0%). In women, the countries with the highest changes in trends were El Salvador (AAPC, −3·9%) and Paraguay (−3·8%), while those with the smallest changes were Panama (AAPC, −0·5%) and Cuba (AAPC, −0·8%).

In the older age group (≥50 years), all ASMRs in men had significant downward trends (supplementary table 2), with the most remarkable changes in Costa Rica and Puerto Rico (AAPCs, −3·7% in both countries). For women, ASMRs are significantly declining in most countries, except for Panama and Puerto Rico. In contrast, widely variable trends were observed in the young group (25-49 years) (Supplementary Table 3). In men, AAPCs ranged from −3·8% in Guatemala to 0·2% in the United States. In women, AAPCs ranged from −3·9% in El Salvador to 2·1% in Panama. Male-to-female ratios for the most recent year declined for some countries (supplementary table 3), but the results were not statistically significant (*p*-values >0.05).

According to the age-period-cohort predictions for 2030, in almost all countries there should be a gastric cancer mortality reduction in men, with an increase in population size and a decrease in change due to risk (Table 1). In women, there would also be a mortality reduction from gastric cancer in most countries (Table 2). However, in Puerto Rico and El Salvador, the reduction in the risk of death would exceed the increase due to changes in the population size and structure.

**Table 1:** Number of gastric cancer deaths, age-standardized mortality rates, and percentage change in cases due to population growth and risk among Hispanic/Latino men in the United States, Latin America, and the Caribbean, 2017 and predicted 2030.^a^

Country (or territory)	Male population aged 25 and older (annual million)		Number of deaths in men aged 25 and older		Age-standardized mortality rates		Total change, %	Change due to population, %	Change due to risk, %
	2017	2030	2017	2030	2017	2030			
Argentina	12·8	14·6	1898	2162	13·6	12·3	15·5	29·7	−14·2
Brazil	65·0	73·7	9188	12575	15·6	13·7	36·9	70·4	−33·5
Chile	6·2	6·6	2191	2480	31·8	23·9	13·7	66·0	−52·3
Colombia	14·8	17·1	3188	4892	24·3	23·3	57·6	79·6	−22·0
Costa Rica	1·6	1·8	398	585	24·3	22·2	48·8	76·0	−27·2
Cuba	4·0	4·1	560	578	10·0	7·8	8·5	34·3	−25·8
Ecuador	4·7	5·8	953	1029	22·0	15·1	14·1	70·4	−56·3
El Salvador	1·5	1·8	221	251	14·2	13·1	7·7	32·9	−25·2
Guatemala	3·4	5·3	558	555	19·6	12·6	−5·7	64·9	−70·6
Mexico	34·8	41·3	3282	4108	10·1	9·1	31·1	51·5	−20·4
Nicaragua	1·6	2·0	186	276	15·7	14·8	51·0	65·9	−14·9
Panama	1·2	1·4	184	281	13·3	15·2	41·8	65·4	−23·6
Paraguay	1·9	2·3	154	195	9·4	8·5	30·7	57·9	−27·2
Peru	9·9	11·0	1536	1908	18·4	13·2	27·1	86·0	−58·9
Puerto Rico	0·9	1·0	102	127	6·2	6·2	8·3	31·5	−23·2
Uruguay	1·0	1·1	204	260	15·2	15·6	7·0	23·4	−16·4
United States	16·7	23·3	1059	1642	7·0	6·4	69·1	92·1	−23·0
Venezuela	7·7	9·9	1107	1289	16·4	12·1	16·5	60·6	−44·0

a Age-standardized rate represents the mid-point for the projected period 2028–2032.

**Table 2:** Number of gastric cancer deaths, age-standardized mortality rates, and percentage change in cases due to population and risk among Hispanic/Latino women in the United States, Latin America, and the Caribbean, 2017 and predicted 2030.^a^

Country (or territory)	Female population aged 25 and older (annual million)		Number of deaths in women aged 25 and older		Age-standardized mortality rates		Total change, %	Change due to population, %	Change due to risk, %
	2017	2030	2017	2030	2017	2030			
Argentina	14·2	16·1	1087	1252	5·7	5·4	17·6	27·2	−9·6
Brazil	70·2	79·9	5082	7399	6·7	6·7	45·0	64·1	−19·1
Chile	6·6	7·0	1106	1198	11·8	9·9	8·6	47·4	−38·8
Colombia	16·1	18·5	1991	2849	12·1	11·7	46·5	77·8	−31·3
Costa Rica	1·6	1·9	252	321	13·3	11·0	34·8	74·9	−40·1
Cuba	4·1	4·2	334	474	5·2	5·3	33·9	39·9	−6·0
Ecuador	4·9	6·0	654	759	13·2	10·0	11·7	71·4	−59·7
El Salvador	2·0	2·3	177	158	8·4	5·0	−19·2	51·7	−70·9
Guatemala	3·9	6·4	620	699	17·2	12·3	1·4	69·7	−68·3
Mexico	38·6	45·6	2863	3786	7·7	7·0	38·0	58·9	−20·9
Nicaragua	1·8	2·2	118	195	7·6	8·2	52·9	69·7	−16·8
Panama	1·2	1·5	118	141	9·3	7·1	23·9	68·1	−44·2
Paraguay	1·8	2·3	87	111	5·2	4·7	32·1	57·9	−25·8
Peru	9·9	11·5	1103	1687	13·5	10·2	25·6	73·0	−47·4
Puerto Rico	1·1	1·2	64	78	2·8	2·6	−5·3	33·8	−39·1
Uruguay	1·2	1·3	141	162	7·0	6·5	8·8	16·9	−8·1
United States	16·6	23·1	812	1314	4·4	4·7	77·8	80·9	−3·1
Venezuela	8·3	10·6	720	764	8·6	6·0	6·7	61·2	−54·4

a Age-standardized rate represents the mid-point for the projected period 2028–2032.

## Discussion

Hispanics/Latinos have low-to-moderate gastric cancer mortality rates. Our study represents the most comprehensive analysis of gastric cancer mortality in these populations. Although there was a substantial variation in ASMRs across countries (up to ∼4-fold overall), we found that in all Hispanic/Latino populations in the United States, Latin America, and the Caribbean overall gastric cancer mortality has declined in the last two decades. Notably, mortality rates are declining in older populations but stable or increasing in a few groups of individuals aged <50 years. Moreover, in these young groups, the classic male predominance of gastric cancer seems to be changing in some countries.

The most common (>90%) anatomical subtype of gastric cancer among Hispanic/Latino populations in the United States and Latin America is *H. pylori*-associated noncardia.^10^, ^11^, ^12^ Declining mortality rates of gastric cancer in older Hispanics/Latinos may reflect decreases in incidence related to a reduction in *H. pylori* acquisition during childhood as well as favorable changes in other risk factors. However, the reasons for discrepant trends in younger individuals are unclear. We and others have reported that the incidence of gastric cancer (mainly noncardia) appears to be leveling off or even increasing among young birth cohorts in the United States and other Western countries,^13^, ^14^, ^15^ potentially unrelated to *H. pylori* infection. In particular, our analysis shows a modest rising in noncardia cancer incidence among United States Hispanics/Latinos aged <50 years (estimated annual percentage change for men and women combined, 0·44%; p<0.05).^15^ In the United States, *H. pylori* seroprevalence among Hispanics/Latinos remains high (57%) and differed significantly by background and nativity.^16^ Studies of *H. pylori* infection in Hispanics/Latinos in Latin America show a sustained prevalence higher than 50%.^17^ In addition, *H. pylori* infection resistance to antibiotics is a growing problem in the Americas.^18^^,^^19^ The control of *H. pylori* infection is a cornerstone to gastric cancer risk reduction, which in turn would decrease mortality. Several randomized trials have shown that *H. pylori* eradication significantly reduces gastric cancer incidence and mortality.^20^ Also, *H. pylori* test-and-treat strategies targeting adults at moderate-to-high gastric cancer risk are likely to be cost-effective.^21^

In most of the Americas, gastric cancer incidence and mortality are similar because of a lack of screening and limited successful treatment options. Unfortunately, diagnosis of gastric cancer at advanced-stage is common in Hispanic/Latino populations, particularly in Latin America where access to specialized oncology centers is in short supply.^22^ Besides incidence, mortality trends are affected by changes in survival. A few studies have addressed gastric cancer survival in Hispanics/Latinos. In line with delayed diagnosis, a population-based study in Colombia showed a low 5-year overall survival (16% for men and 18% for women) for the period 1994–2004.^23^ In the US, Liu et al., reported slightly higher 5-year survival estimates of 37·6% for Hispanics in Florida and 33·5% for Hispanics in SEER for the 2006-2016 period.^24^

Mortality differences across countries could be explained by variations in prevention strategies, access to healthcare services, health insurance coverage, and patient care practices. Except for Costa Rica, no other country in this study has implemented secondary prevention programs.^25^ Gastric cancer is a multistep process with well-defined premalignant histological stages that allow a wide window of intervention. As recommended by international guidelines,^26^ endoscopy surveillance of patients with severe gastric atrophy could provide an opportunity to control gastric cancer. In particular, high-risk countries in the Latin American region should consider a consistent follow-up of patients with advanced intestinal metaplasia (i.e., corpus extension). This secondary prevention strategy would allow identification of treatable gastric lesions associated with good prognosis. Future prospective studies should define the optimal time for endoscopy and the identification of non-invasive biomarkers of severe gastric atrophy that can be combined with circulating pepsinogens.

As expected, our study showed that South and Central American countries, Chile, Guatemala, Costa Rica, Colombia, Ecuador, and Peru in particular, had the highest mortality rates of gastric cancer, while North American countries and those in the Caribbean had the lowest rates. Studies in the Pacific rim and Andean regions have suggested that a geographic clustering, coupled with low-economic income settings, poor education, and limited access to healthcare, creates an environment that allows the spread of risk factors that limit the control of mortality from gastric cancer.^27^

Most countries in our analysis had mortality reductions between 2 to 3% per year during the study period, and the projected ASMR for 2030 would yield similar or even smaller declines. These estimates are lower than those observed in other populations without screening programs. Major European countries had downward trends between 3 to 4% for the 1980–2005 period,^28^ probably related to an earlier reduction in gastric cancer risk factors and better access to health care services.

Our analysis confirmed an overall male predominance in gastric cancer mortality. Although the reasons underlying this sex difference is likely complex and multifactorial, men may have a higher prevalence of gastric cancer risk factors (i.e., poor hygiene, lower consumption of fruits or vegetables, and higher tobacco consumption)^29^ that are being reduced over time. Nevertheless, while women are more attentive to health-related information and behaviors that potentially affect health, men are prone to avoid healthcare visits, which increases the risk of detecting gastric cancer in late stages and, thus, the mortality risk.

The ∼60 million Hispanics/Latinos in the United States represent 18% of the country's total population in 2020. This population group deserves special public health attention given its aging and rapid growth. The United States Hispanics/Latinos represent a heterogeneous population of United States- and foreign-born immigrants mainly from Mexico, Central America, and the Caribbean. Our study found that for 2017 ASMRs in the United States were lower (7·0 per 100 000 for men and 4·4 for women) than most Latin American countries. Mortality in United States Hispanics/Latinos could be influenced by migration patterns, entailing a socio-cultural behavioral difference between immigrants and non-Hispanic whites. These disparities of cancer mortality are important hurdles to reduce the burden of gastric cancer for which population tailored approaches are needed, as well as increasing access to healthcare services.

This study has the usual caveats of a cancer registry analysis, such as missing data, lack of specific information on tumor characteristics (i.e., histology and anatomical subsite), limited individual-level information, and variation in death registration completeness and quality. Ongoing initiatives to establish population-based cancer incidence registries in the Central America Four region (comprising Guatemala, Honduras, El Salvador, and Nicaragua)^30^ are expected to improve cancer information and amend for the potential underreporting of available mortality data. Unfortunately, we could not retrieve mortality information from some countries in the region, including Honduras and Bolivia. Torres *et al*. reported an ASMR of 15·1 per 100 000 for men and 13 for women in Bolivia.^27^ The GLOBOCAN 2020 (Latin America Hub) ASMRs for men (ages ≥25 years) are 20·5 and 14·6 for Honduras and Bolivia, respectively. The absence of gastric cancer mortality data could limit public health interventions in these least developed Latin American countries.

In conclusion, although Hispanic/Latino populations in the United States, Latin America, and the Caribbean have overall downward mortality trends of gastric cancer and ASMRs are predicted to decrease further, this geographic region still has one of the highest mortality rates globally. Future research should explore factors underlying the stable or increasing mortality rates in some young population groups. Further surveillance of age-specific mortality and incidence trends will be informative. Our findings emphasize the need for timely and effective control and prevention strategies in these populations to ensure that these unfavorable trends do not persist.

## Contributors

JS Torres-Roman and MC Camargo conceived the study. JS Torres-Roman and P Guerra-Canchari extracted and organized the mortality data. DLB de Souza provided guidance on methods. JS Torres-Roman, CS Alvarez, C Alves Santos, SCM Soares and DLB de Souza analyzed the data. B Valcarcel was responsible for data visualization. JS Torres-Roman, CS Alvarez, JF Martinez-Herrera, and MC Camargo wrote the original draft. All authors reviewed and edited the manuscript. All authors had full access to the data and accept responsibility to submit for publication.

## Data sharing statement

Data are available in an open access repository and can be accessed and downloaded at: https://www-dep.iarc.fr/whodb/whodb.htm.

## Declaration of interests

All authors declare that they have no conflict of interest with this study.

## Appendix Supplementary materials

Image, application 1
